# Supplementary material for: Identifying novel inhibitors against drug-resistant mutant CYP-51 Candida albicans: A computational study to combat fungal infections
Source: PLoS One. 2025 Mar 4;20(3):e0318539. doi: 10.1371/journal.pone.0318539 (PMC11878927; doi:10.1371/journal.pone.0318539)
Supplement: S1 Table — (DOCX) [file pone.0318539.s001.docx]

**S1 Table.** List of novel inhibitors against Candida albicans assessed through PubChem along with the 2D and 3D molecular conformations of the compounds prepared using ChemDraw Professional 16.0.

| **Codes** | **Novel inhibitor** | **2D** | **3D** | **SMILES** |
| --- | --- | --- | --- | --- |
| **CP-1** | [6-[4-[4-[2-(aminomethyl)-1H-imidazol-5-yl]phenyl]phenyl]-1H-benzimidazol-2-yl]methanamine |  | 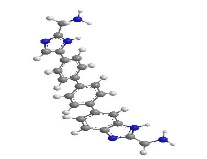 | C1=CC(=CC=C1C2=CC=C(C=C2)C3=CN=C(N3)CN)C4=CC5=C(C=C4)N=C(N5)CN |
| **CP-2** | 3,5-bis[4-(3-hydroxypropoxy)phenyl]phenol |  | 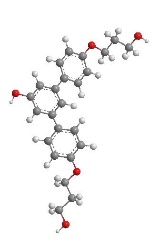 | C1=CC(=CC=C1C2=CC(=CC(=C2)O)C3=CC=C(C=C3)OCCCO)OCCCO |
| **CP-3** | (4-[2-[4-[(2-aminoethylamino)methyl]phenyl]ethynyl]-N-[(2S)-3-amino-1-(hydroxyamino)-1-oxopropan-2-yl]benzamide;methane) |  | 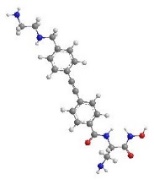 | C.C1=CC(=CC=C1CNCCN)C#CC2=CC=C(C=C2)C(=O)N[C@@H](CN)C(=O)NO |
| **CP-4** | 7-[(1E,4E)-5-(5-carbamimidoyl-1H-indol-2-yl)penta-1,4-dienyl]quinoline-2-carboximidamide |  | 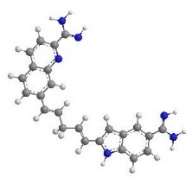 | C1=CC(=CC2=C1C=CC(=N2)C(=N)N)/C=C/C/C=C/C3=CC4=C(N3)C=CC(=C4)C(=N)N |
| **CP-5** | 3-[3-fluoro-4-[(E)-3-hydroxyprop-2-enoxy]phenyl]-5-[4-(2-hydroxyethoxy)phenyl]phenol |  | 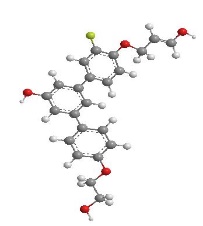 | C1=CC(=CC=C1C2=CC(=CC(=C2)O)C3=CC(=C(C=C3)OC/C=C/O)F)OCCO |
| **CP-6** | 2-[4-[3-fluoro-4-[4-(1-hydroxypropan-2-yloxy)phenyl]phenyl]phenoxy]propan-1-ol |  | 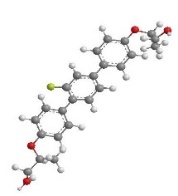 | CC(CO)OC1=CC=C(C=C1)C2=CC(=C(C=C2)C3=CC=C(C=C3)OC(C)CO)F |
| **R* (CCL)** | 4-[4-[4-[4-[[(3R,5R)-5-(2,4-difluorophenyl)-5-(1,2,4-triazol-1-ylmethyl)oxolan-3-yl]methoxy]phenyl]piperazin-1-yl]phenyl]-2-[(2S,3S)-2-hydroxypentan-3-yl]-1,2,4-triazol-3-one |  | 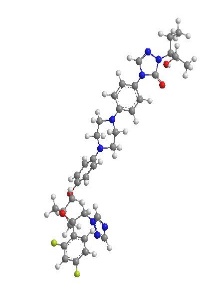 | CC[C@@H]([C@H](C)O)N1C(=O)N(C=N1)C2=CC=C(C=C2)N3CCN(CC3)C4=CC=C(C=C4)OC[C@H]5C[C@](OC5)(CN6C=NC=N6)C7=C(C=C(C=C7)F)F |
