# Supplementary material for: Identifying novel inhibitors against drug-resistant mutant CYP-51 Candida albicans: A computational study to combat fungal infections
Source: PLoS One. 2025 Mar 4;20(3):e0318539. doi: 10.1371/journal.pone.0318539 (PMC11878927; doi:10.1371/journal.pone.0318539)
Supplement: S2 Table — (DOCX) [file pone.0318539.s002.docx]

**S2 Table.** Protein structure-based prediction.

| **CYP-51 (Y132H)** | **Score** | **Prediction** |
| --- | --- | --- |
| **I-Mutant** | −2.19 kcal/mol | Decrease |
| **CUPSAT** | −10.38 kcal/mol | Destabilizing |
| **mCSM** | −1.702 kcal/mol | Destabilizing |
| **DUET** | −1.597 kcal/mol | Destabilizing |
| **SDM** | −0.67 kcal/mol | Destabilizing |
