# Supplementary material for: Identifying novel inhibitors against drug-resistant mutant CYP-51 Candida albicans: A computational study to combat fungal infections
Source: PLoS One. 2025 Mar 4;20(3):e0318539. doi: 10.1371/journal.pone.0318539 (PMC11878927; doi:10.1371/journal.pone.0318539)
Supplement: S3 Table — (DOCX) [file pone.0318539.s003.docx]

**S3 Table.** The quality of crystal structure mutated CYP-51 (Y132H) structure.

| **RAMACHANDRAN PLOT** | **PROCHCK** | **ERRAT** |
| --- | --- | --- |
| 94.6% | Pass | 90.1 |
