# Supplementary material for: Identifying novel inhibitors against drug-resistant mutant CYP-51 Candida albicans: A computational study to combat fungal infections
Source: PLoS One. 2025 Mar 4;20(3):e0318539. doi: 10.1371/journal.pone.0318539 (PMC11878927; doi:10.1371/journal.pone.0318539)
Supplement: S4 Table — (DOCX) [file pone.0318539.s004.docx]

**S4 Table** Physiochemical properties of protein with electrostatic potential of ligand CP-3.

| **Ligand** | **HOMO**  **(a.u)** | **LUMO (a.u)** | **Dipole moment**  **(a.u.)** | **Energy gap (ΔEGap)**  **(a.u.)** | **Ionization Potential (a.u)** | **Electronic affinity (a.u)** | **Electronegativity**  **(a.u.)** | **Electrochemical potential μ (a.u.)** | **Hardness (a.u.)** | **Softness (a.u.)** | **Electrophilicity (a.u.)** |
| --- | --- | --- | --- | --- | --- | --- | --- | --- | --- | --- | --- |
| **CP-3** | -0.20744 | -0.06208 | 5.569 um | 0.14536 | 0.20393 | 0.04268 | 0.123305 | -0.123305 | 0.080625 | 12.405 | 0.09422 |
