# Supplementary material for: Identifying novel inhibitors against drug-resistant mutant CYP-51 Candida albicans: A computational study to combat fungal infections
Source: PLoS One. 2025 Mar 4;20(3):e0318539. doi: 10.1371/journal.pone.0318539 (PMC11878927; doi:10.1371/journal.pone.0318539)
Supplement: S5 Table — (DOCX) [file pone.0318539.s005.docx]

**S5 Table:** Per-residue scores of lead compound CP-3 complexed with mutated protein CYP-51.

| **Residue** | **van der Waals** | **Electrostatic** | **Polar Solvation** | **Non-Polar Solv.** | **TOTAL** |
| --- | --- | --- | --- | --- | --- |
|  | Avg. | Avg. | Avg. | Avg. |  |
| PHE 14 | -0.7584 | 0.1631 | -0.074 | -0.13859136 | -0.80789136 |
| ALA 17 | -1.4301 | -0.4504 | 0.3923 | -0.11670552 | -1.60490552 |
| ALA 18 | -0.7044 | -0.0972 | 0.3628 | -0.11115072 | -0.54995072 |
| GLY 21 | -1.1433 | -0.3802 | 0.737 | -0.0965808 | -0.8830808 |
| LEU 43 | -0.8239 | -0.2942 | -0.0336 | -0.09355104 | -1.24525104 |
| LEU 44 | -0.8192 | -0.2579 | 0.2013 | -0.08466768 | -0.96046768 |
| TYR 74 | -1.4283 | -0.6081 | 2.1569 | -0.18843624 | -0.06793624 |
| LEU 77 | -0.9152 | 0.036 | -0.3647 | -0.0891252 | -1.3330252 |
| PHE 82 | -1.408 | -0.0946 | -0.1051 | -0.16483608 | -1.77253608 |
| ILE 87 | -2.1268 | -0.8248 | 0.466 | -0.25057656 | -2.73617656 |
| TYR 88 | -1.441 | -0.1615 | 0.8861 | -0.20526264 | -0.92166264 |
| PRO 186 | -2.4642 | -0.1695 | 0.4303 | -0.28770264 | -2.49110264 |
| PHE 189 | -1.3336 | -0.2983 | 0.5968 | -0.1141992 | -1.1492992 |
| LEU 256 | -0.503 | 0.1328 | -0.2326 | -0.01390608 | -0.61670608 |
| GLY 259 | -1.0411 | -1.6708 | 1.3731 | -0.08101512 | -1.41981512 |
| ILE 260 | -1.1902 | -0.1359 | 0.054 | -0.05098032 | -1.32308032 |
| GLY 263 | -0.6801 | -0.1287 | 0.2995 | -0.12548736 | -0.63478736 |
| HIE 333 | -1.3112 | 0.367 | -0.0036 | -0.08845704 | -1.03625704 |
| PHE 336 | -1.1827 | -0.3503 | 0.4903 | -0.11630736 | -1.15900736 |
| HIE 424 | -0.7058 | -0.1014 | 0.6816 | -0.0774756 | -0.2030756 |
| CYS 426 | -0.8999 | -0.0386 | 0.4043 | -0.161226 | -0.695426 |
| ILE 427 | -0.8434 | -0.3985 | 0.942 | -0.10453968 | -0.40443968 |
| TYR 461 | -0.6158 | -1.0233 | 1.2226 | -0.02167776 | -0.43817776 |
| SER 462 | -1.4656 | 0.0442 | 0.7188 | -0.20033064 | -0.90293064 |
| SER 463 | -0.9639 | -0.9932 | 1.3405 | -0.0157644 | -0.6323644 |
| MET 464 | -2.9243 | -0.5678 | 1.9215 | -0.28404144 | -1.85464144 |
