# Supplementary material for: Identifying novel inhibitors against drug-resistant mutant CYP-51 Candida albicans: A computational study to combat fungal infections
Source: PLoS One. 2025 Mar 4;20(3):e0318539. doi: 10.1371/journal.pone.0318539 (PMC11878927; doi:10.1371/journal.pone.0318539)
Supplement: S6 Table — (DOCX) [file pone.0318539.s006.docx]

**S6 Table:** Per-residue scores of R* compound (CCL) complexed with mutated protein CYP-51.

| **Residue** | **van der Waals** | **Electrostatic** | **Polar Solvation** | **Non-Polar Solv.** | **TOTAL** |
| --- | --- | --- | --- | --- | --- |
|  | Avg. | Avg. | Avg. | Avg. |  |
| LEU44 | -0.9243 | -0.3092 | 0.3753 | -0.10791792 | -0.96612 |
| TYR74 | -1.7154 | -0.7277 | 1.8549 | -0.24064272 | -0.82884 |
| THR78 | -0.0755 | 0.0476 | -0.063 | -0.00943848 | -0.10034 |
| PHE82 | -0.1024 | 0.0558 | -0.0043 | -0.02499264 | -0.07589 |
| ILE87 | -0.0895 | -0.059 | -0.0078 | -0.00641808 | -0.16272 |
| TYR88 | -0.3391 | -0.4291 | 0.7143 | -0.12517992 | -0.17908 |
| PHE184 | -0.3316 | 0.0228 | 0.1082 | -0.06783552 | -0.26844 |
| THR185 | -0.0786 | -0.0966 | -0.1589 | 0 | -0.3341 |
| PRO186 | -0.8124 | -0.288 | 0.5307 | -0.11369304 | -0.68339 |
| ILE187 | -0.1391 | 0.0291 | -0.0334 | -0.00028656 | -0.14369 |
| ASN188 | -0.0761 | 0.1332 | -0.4736 | 0 | -0.4165 |
| PHE189 | -2.5509 | 0.3907 | 0.121 | -0.25925472 | -2.29845 |
| VAL190 | -0.5083 | 0.0706 | -0.1666 | -0.02571552 | -0.63002 |
| GLY263 | -0.1336 | -0.9131 | 0.3417 | -0.06119064 | -0.76619 |
| PRO331 | -0.1433 | -0.0483 | 0.1222 | 0 | -0.0694 |
| LEU332 | -2.101 | 0.0283 | -0.0516 | -0.23352192 | -2.35782 |
| HIE333 | -1.0795 | -0.0038 | 0.1522 | -0.07021152 | -1.00131 |
| SER334 | -1.2798 | -0.1219 | 1.0439 | -0.15667128 | -0.51447 |
| ILE335 | -0.9607 | -0.0323 | -0.0579 | -0.08659512 | -1.1375 |
| PHE336 | -1.6471 | -0.0739 | 0.331 | -0.17871984 | -1.56872 |
| MET464 | -1.8071 | -1.5545 | 1.1794 | -0.23641344 | -2.41861 |
| VAL465 | -0.2416 | -0.081 | -0.114 | -0.00027144 | -0.43687 |
