# Supplementary material for: Identifying novel inhibitors against drug-resistant mutant CYP-51 Candida albicans: A computational study to combat fungal infections
Source: PLoS One. 2025 Mar 4;20(3):e0318539. doi: 10.1371/journal.pone.0318539 (PMC11878927; doi:10.1371/journal.pone.0318539)
Supplement: S1 Fig — (DOCX) [file pone.0318539.s007.docx]

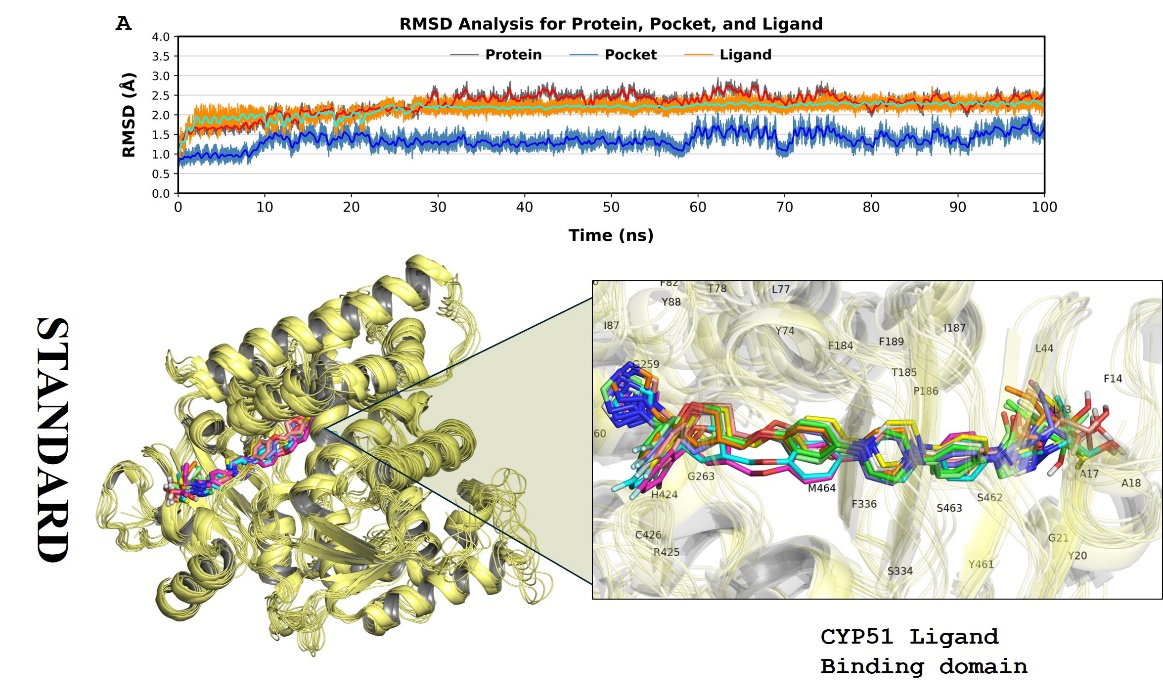


**S1 Fig:** Molecular dynamics (MD) simulation analysis of the mutated CYP-51 (Y132H) complexes representing time-dependent-RMSDs of protein Cα atoms, pocket, and the ligand relative to the protein along with the visual representation of R* (CCL) docked complex showing ligand binding domain.
