# Supplementary material for: Identifying novel inhibitors against drug-resistant mutant CYP-51 Candida albicans: A computational study to combat fungal infections
Source: PLoS One. 2025 Mar 4;20(3):e0318539. doi: 10.1371/journal.pone.0318539 (PMC11878927; doi:10.1371/journal.pone.0318539)
Supplement: S2 Fig — (DOCX) [file pone.0318539.s008.docx]

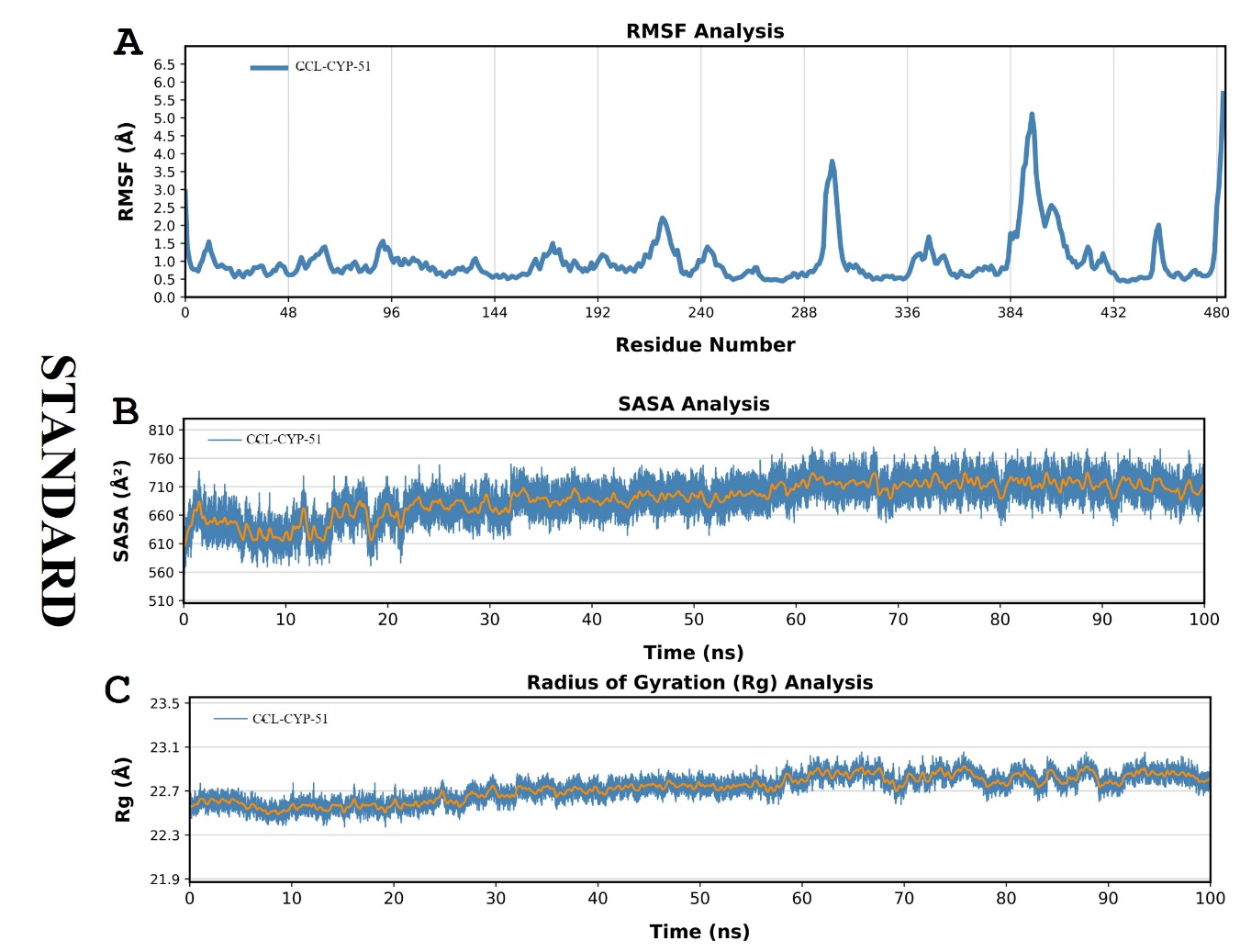
**S2 Fig:** Graphical representation of RMSF, SASA, and Rg of the R* (CCL) complexed with mutated target protein CYP-51 during simulation trajectory
