# Supplementary material for: Identifying novel inhibitors against drug-resistant mutant CYP-51 Candida albicans: A computational study to combat fungal infections
Source: PLoS One. 2025 Mar 4;20(3):e0318539. doi: 10.1371/journal.pone.0318539 (PMC11878927; doi:10.1371/journal.pone.0318539)
Supplement: S3 Fig — (DOCX) [file pone.0318539.s009.docx]

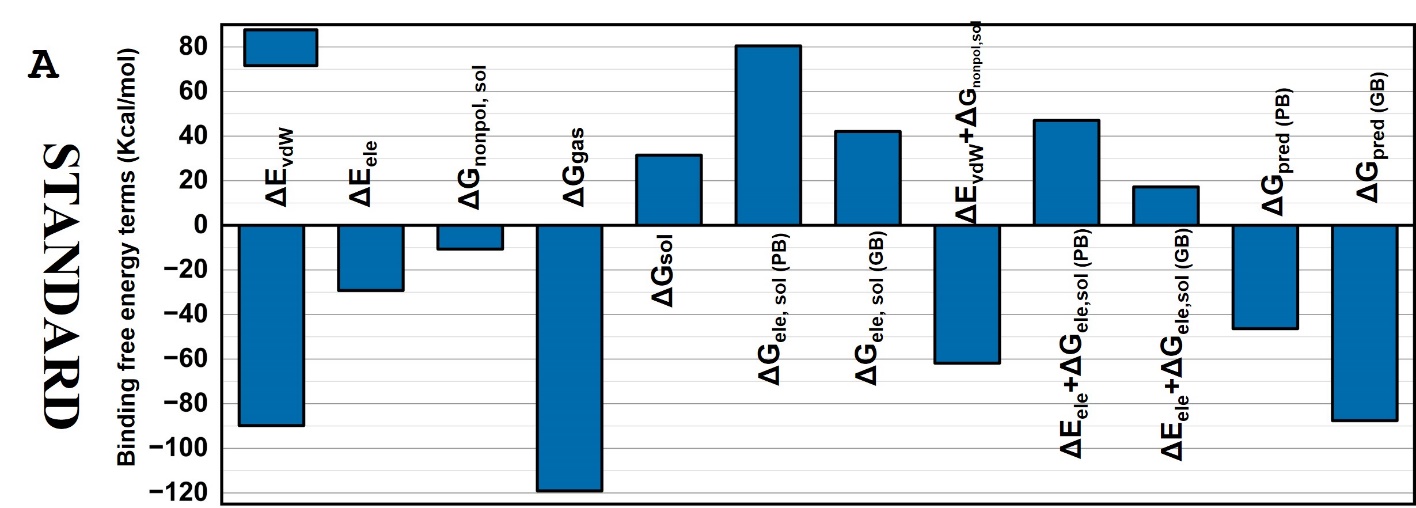


**S3 Fig:** Plot representing the free binding energies of CYP-51– R* (CCL) complex during simulation trajectory.
