# Supplementary material for: Identifying novel inhibitors against drug-resistant mutant CYP-51 Candida albicans: A computational study to combat fungal infections
Source: PLoS One. 2025 Mar 4;20(3):e0318539. doi: 10.1371/journal.pone.0318539 (PMC11878927; doi:10.1371/journal.pone.0318539)
Supplement: Attachment — Submitted filename: Response Letter.docx [file pone.0318539.s010.docx]

**Response Letter to the Reviewer Comments**

We sincerely thank you and the reviewers for your detailed and constructive feedback on our manuscript. We have carefully addressed all comments and revised the manuscript accordingly. Below, we provide a detailed point-by-point response to each comment.

**Reviewer 1 Comments:**

**Comment 1:** Avoid abbreviations in the abstract.

**Response:** We appreciate the reviewer's suggestion and have revised the abstract to eliminate abbreviations.

**Comment 2:** Avoid abbreviations in keywords

**Response:** We appreciate the respectable reviewer’s recommendation and have accordingly removed all abbreviations from the keywords section.

**Comment 3:** Please indicate the full name of each abbreviation when it first appears in the draft.

**Response:** We thank the reviewer for this valuable comment. We have ensured that all abbreviations are fully defined when they first appear in the manuscript.

**Comment 4:** Enhance the introduction section of your study by incorporating the specified articles to compare your results with those of other studies

**Response:** We are grateful for this insightful suggestion. The introduction section has been updated to incorporate the specified studies. Furthermore, we have added multiple studies in the discussion section to compare our results with previous studies.

**Comment 5:** 2D structures in Table One should all be in the same size and style

**Response:** We express our gratitude for the reviewer's observation. We have standardized the size and style of all 2D structures in Table One to ensure consistency.

**Comment 6:** The titles of all figures should be written more scientifically

**Response:** We appreciate the reviewer’s suggestion. The titles of all figures have been revised to adhere to a more scientific and precise format.

**Comment 7:** Use more recent references

**Response:** We express our gratitude to the reviewer for highlighting this issue. We have updated the reference list with more recent and relevant studies to enhance the manuscript.

**Reviewer 2 Comments:**

**Comment 1:** Authors should elaborate more, providing more details on the target topology and pocket description prior to presenting the docking findings.

**Response:** We thank the reviewer for your valuable suggestion. We have revised the manuscript to include a more detailed description of the target protein in the result section, ensuring a clearer context before presenting the docking results.

**Comment 2:** Positive control reference compound should be adopted throughout the study to provide significance for the obtained computational findings in terms of relevant biological translation.

**Response:** We appreciate the esteemed reviewer’s constructive feedback. The manuscript has been revised to incorporate an analysis of the standard reference compound, highlighting its significance across all investigated parameters. It should be taken into account that the molecular docking and ADMET profiling all-encompassed that analysis for the reference compound. Additionally, the comparative analysis was further incorporated in the MD simulations, presented in supplementary files.

**Comment 3:** Authors should elaborate more on the ligand-target interaction patterns Bonding should be annotated in terms of both the bond distances and angles. Specially for Hydrogen bonding, this type of compound-protein polar interaction should be presented within hydrogen bond distances as well as bond angles since hydrogen bond depend on both. Authors should mention the Hydrogen bond angles as well as their distances, since the strength of hydrogen bonding is based on both parameters in a way to ensure the adequacy of optimum hydrogen bonding.

**Response:** We express our gratitude to the esteemed reviewer for this technical observation. We have conducted a revised binding interaction analysis for all compounds, including the reference compound, incorporating detailed annotations of bond distances and angles for each interaction. Particular emphasis has been placed on hydrogen bonds to ensure an accurate representation of their strength and adequacy.

**Comment 4:** The author provided dissection of the free binding energies calculations estimated from the MD simulation trajectories in terms of ∆G electrostatic, ∆G van der Waal, ∆G polar solvation, ∆G non-polar SASA solvation,…) to identify the dominant energy terms that can guide further ligand optimization and development.
Further, the total free binding energies for compound-target complex should be broken down to the target residue energy contribution to highlight which residues impose the great impact on ligand binding for future targeting and lead optimization.

**Response:** We sincerely appreciate your insightful suggestion. We have added target residue energy contribution in the result section, highlighting the impact of residues on ligand binding.

**Comment 5:** Authors are advised to provide snap shoot of the MD simulated ligand-protein complex at different time intervals (example 0ns, 50ns, 100ns, 150ns, and/or 200ns) to track the main conformational/orientation changes for both the compound and surrounding residues over time.

**Response:** We thank the reviewer for this excellent suggestion. We have added snapshots of the MD-simulated complexes at specified time intervals in the result section to visualize conformational and orientation changes of the ligand and surrounding residues over time.

**Comment 6:** Based on the study results, what are the take-away messages. Authors are advised to highlight the suggested structural modifications that would improve the compound’s biological activities based on the in silico findings. These insights would be beneficial for guiding further lead optimization and development.

**Response:** We appreciate the suggestion; we have improvised and discussed in the conclusion section by focusing on the key residues for structural and chemical modification of the compound to enhance the inhibitory efficacy of the compound for therapeutic purposes.

**Comment 7:** Finally, concerning the conclusion, authors are advised to elaborate more on the future of this work? Will you broaden the scope to another drug target? What are the study limitations and what approaches could be conducted to further address them?

**Response:** We are grateful for the thoughtful insights. We have expanded the conclusion to discuss the potential future directions of this research, including broadening the scope to other drug targets. Additionally, we have outlined the study’s limitations and proposed approaches to address them in subsequent investigations.
